# Risk of biliary tract disease in living liver donors: A population-based cohort study

**DOI:** 10.1371/journal.pone.0230840

**Published:** 2020-03-30

**Authors:** Shih-Yi Lin, Cheng-Li Lin, Wu-Huei Hsu, I-Kuan Wang, Cheng-Chieh Lin, Long-Bing Jeng, Chia-Hung Kao

**Affiliations:** 1 Graduate Institute of Clinical Medical Science, College of Medicine, China Medical University, Taichung, Taiwan; 2 Division of Nephrology and Kidney Institute, China Medical University Hospital, Taichung, Taiwan; 3 Management Office for Health Data, China Medical University Hospital, Taichung, Taiwan; 4 College of Medicine, China Medical University, Taichung, Taiwan; 5 Division of Pulmonary and Critical Care Medicine, China Medical University Hospital and China Medical University, Taichung, Taiwan; 6 Department of Family Medicine, China Medical University Hospital, Taichung, Taiwan; 7 Division of Surgery, China Medical University Hospital, Taichung, Taiwan; 8 Department of Nuclear Medicine and PET Center, and Center of Augmented Intelligence in Healthcare, China Medical University Hospital, Taichung, Taiwan; 9 Department of Bioinformatics and Medical Engineering, Asia University, Taichung, Taiwan; Indiana University, UNITED STATES

## Abstract

**Background & aims:**

Whether living liver donors have a higher risk of biliary tract disease compared with non-donors remains unknown.

**Methods:**

Data were collected from the Taiwan Longitudinal Health Insurance Database for the 2003–2011 period. The study cohort comprised 1,446 patients aged ≥ 18 years who had served as living liver donors. The primary outcome was the incidence of biliary tract disease. Cox proportional hazards modeling was used to determine the hazard ratios.

**Results:**

The incidence density rate of biliary tract disease was 13.9-fold higher in the liver donor (LD) cohort than in the non-LD cohort (10.2 vs. 0.71 per 1,000 person-years), with an adjusted hazard ratio (HR) of 14.2 (95% confidence interval [CI] = 7.73–26.1). Stratified by comorbidity, the relative risk of biliary tract disease was higher in the LD cohort than in the non-LD cohort for both patients with or without comorbidity. The incidence density rate of biliary tract disease was significantly higher in the first 3 years (13.5 per 1,000 person-years in the LD cohort). The highest adjusted HR of biliary tract disease for LD patients compared with the non-LD cohort was 22.4 (95% CI = 10.8–46.1) in the follow-up ≤ 3 years.

**Conclusion:**

Living liver donors had a higher risk of biliary tract disease compared with non-donors.

## Introduction

The first living-donor liver transplantation (LDLT) was successfully performed in 1989 [1]. With the shortage of available cadaveric liver, LDLT has offered a therapeutic solution for hepatic failure [2]. Thousands of LDLTs have been conducted worldwide, especially in developed countries [3–5]. A living liver donation requires right- or left-lobe resection from donors. Because it is larger than the left lobe, the right lobe is more adapted to the metabolic demands of recipients [6]. However, right-lobe donation carries an approximately 0.5% higher risk of mortality [7]. In addition, the morbidity of donors following liver donation remains a concern. Because living donors are defined as healthy and qualified for liver donation, morbidity in donors should be reduced to as near zero as possible. Therefore, studying the complications that arise among living liver donors is of value. Numerous studies have investigated aspects of living liver donors, including volumetric and functional recovery [8], morbidity [9], and laboratory testing [10,11]. However, most studies investigating complications among living liver donors were single-center based [8,12–14] or multicenter based studies [9,15,16]. Few population-based cohort studies regarding complications among living liver donors exist. Umeshita et al surveyed the operative morbidity of living liver donors in Japan [3]. Hashikura et al conducted a comprehensive medical review in Japan in 2009 [17]. With more advanced techniques and accumulated experience of living liver donations, new nationwide data regarding complications among living liver donors are necessary. Thus, we used Taiwan’s National Health Insurance Research Database (NHIRD), which comprises records from the population-based medical reimbursement system in Taiwan, as the data source of this study. We investigated whether living liver donors exhibited an increased risk of biliary tract disease.

## Methods

### Data source

The National Health Insurance (NHI) program covers over 99% of Taiwan’s population (23 million) and more than 97% of its health care institutions [18]. The NHIRD was established by the National Health Research Institute (NHRI) and contains claims data from the NHI program from 1996 to 2011. The details of the NHI program and the NHIRD have been previously documented [19,20]. In this retrospective cohort study, the disease history of insured individuals was collected from inpatient data. To protect the patients’ privacy, all personal identification numbers were encrypted by the NHRI before the data were released. The diseases were coded according to the International Classification of Diseases, Ninth Revision, Clinical Modification (ICD-9-CM) diagnosis codes.

### Ethics statement

The NHIRD encrypts patient personal information to protect privacy and provides researchers with anonymous identification numbers associated with relevant claims information, including sex, date of birth, medical services received, and prescriptions. Therefore, patient consent is not required to access the NHIRD. This study was approved to fulfill the condition for exemption by the Institutional Review Board (IRB) of China Medical University (CMUH-104-REC2-115-CR4). The IRB also specifically waived the consent requirement.

### Sampled participants

The liver donor (LD) cohort included new LD patients (ICD-9-CM code V596) from 2003 to 2011, and the index date was set as the initial liver transplant date. Patients with a history of biliary tract disease (ICD-9-CM codes 574, 576) prior to the index date or with missing information regarding age or sex were excluded. The non-LD cohort was randomly identified from the NHIRD during the same period from 2003 to 2011 and frequency matched to the LD cohort by age (every 5 years span), sex, monthly income, and index year, using the same exclusion criteria. All patient data were followed from the index date to the date of diagnosis of biliary tract disease, withdrawal from the NHI program, or the end of 2011, whichever occurred first. The LD cohort and non-LD cohort were matched at a 1:1 ratio based on propensity scores using nearest neighbor matching, initially to the eighth digit and then as needed to the first digit. We used logistic regression to calculate the propensity score for drug status by estimating the assignment probability based on baseline variables, including age, sex, monthly income, and comorbidity of arthropathies and related disorders, dorsopathies, rheumatism, excluding the back, osteopathies, chondropathies, and acquired musculoskeletal deformities, ischemia heart disease, diabetes mellitus, and helicobacter pylori infection.

### Comorbidities

The risk factors for biliary tract disease were identified in all subjects. Arthropathies and related disorders (ICD-9-CM codes 710–719); dorsopathies (ICD-9-CM codes 720–724); rheumatism (excluding the back) (ICD-9-CM codes 725–729); osteopathies, chondropathies, and acquired musculoskeletal deformities (ICD-9-CM codes 730–739); ischemic heart disease (ICD-9-CM codes 410–414); diabetes mellitus (ICD-9-CM code 250); and helicobacter pylori infection (ICD-9-CM code 041.86) were identified according to their diagnoses in the hospitalization records data prior to the index date.

### Statistical analysis

The means and standard deviations (SDs) are provided for continuous variables, and the numbers and percentages are presented for categorical variables. To assess the distribution differences between the LD and non-LD cohorts, Student’s t and chi-square tests were performed for the continuous (age and follow-up time) and categorical variables (age group, sex, monthly income, and comorbidity), respectively. The incidence density of developing biliary tract disease was calculated as the number of biliary tract disease events divided by the sum of the observation time (per 1,000 person-years). Poisson regression was conducted to calculate the incidence rate ratios (IRRs) and the 95% confidence intervals (CIs) of the associations between the risk of biliary tract disease and LD. A multivariable Cox proportional hazards regression analysis was also performed to measure the hazard ratios (HRs) and 95% CIs of biliary tract disease associated with LD, adjusting for age, sex, income, and comorbidities. All analyses were performed using SAS version 9.4 (SAS Institute, Inc., Cary, NC, USA). A *p* of <0.05 was considered statistically significant.

## Results

**Fig 1:**
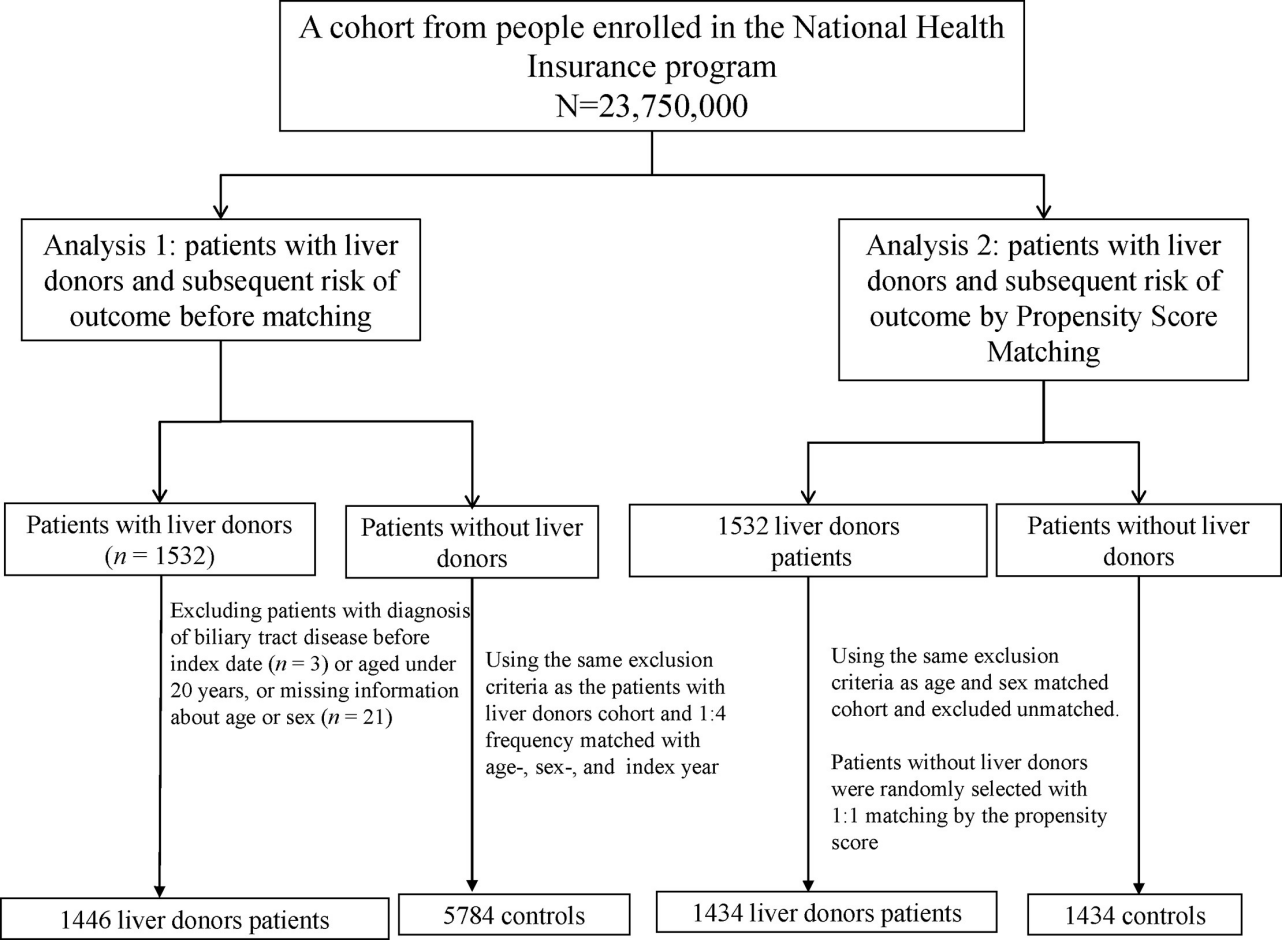
Flowchart of this propensity-matching study.

This study identified 1,446 persons for the LD cohort and 5,784 persons for the non- LD cohort. (Fig 1) Both cohorts were similar in age, sex, and income distributions with a mean age of 32.9 years; nearly 55.3% of the subjects were men with income levels between NT$15,000 and NT$22,799 (46.7%) (Table 1). In addition, 1,434 patients in the LD cohort were matched with 1,434 control patients according to the propensity scores. The LD cohort was more likely to have arthropathies and related disorders; dorsopathies; rheumatism (excluding the back); osteopathies, chondropathies, and acquired musculoskeletal deformities; and helicobacter pylori infection compared with the non-LD cohort. After PS-matched, the two cohorts were more similar in the baseline characteristics.

**Table 1:** Comparison of demographic status and comorbidities between liver donors and non-donors at baseline.

	Age and Sex Matched				*p*-value	Propensity Score Matched				*p*-value
	Non-donors		Donors			Non-donors		Donors		
	(N = 5784)		(N = 1446)			(N = 1434)		(N = 1434)		
	n	%	n	%		n	%	n	%	
**Age, year**					0.99					0.99
≤34	3772	65.2	943	65.2		928	64.7	933	65.1	
35–54	1888	32.6	472	32.6		473	33.0	470	32.8	
≧55	124	2.14	31	2.14		33	2.30	31	2.16	
Mean (SD) ^†^	32.9	9.74	32.9	9.43	0.80	33.1	9.86	32.9	9.45	0.60
Follow-up time, year Mean (SD) ^†^	3.41	1.82	2.86	1.99	<0.001	3.43	1.79	2.87	1.99	0.001
**Sex**					0.99					0.79
Female	2584	44.7	646	44.7		630	43.9	637	44.4	
Male	3200	55.3	800	55.3		804	56.1	797	55.6	
**Monthly income(NTD $)**					0.83					0.96
<15,000	1944	33.6	474	32.8		475	33.1	471	32.9	
15,000–22,799	2658	46.0	675	46.7		660	46.0	668	46.6	
≥22,800	1182	20.4	297	20.5		299	20.9	295	20.6	
**Comorbidity**										
Arthropathies and related disorders	50	0.86	22	1.52	0.02	22	1.53	20	1.39	0.76
Dorsopathies	39	0.67	28	1.94	<0.001	34	2.37	26	1.81	0.30
Rheumatism, excluding the back	44	0.76	24	1.66	0.002	20	1.39	22	1.53	0.76
Osteopathies, chondropathies, and acquired musculoskeletal deformities	37	0.64	50	3.46	<0.001	34	2.37	39	2.72	0.55
Ischemia heart disease	26	0.45	11	0.76	0.14	14	0.98	11	0.77	0.55
Diabetes mellitus	44	0.76	15	1.04	0.30	15	1.05	14	0.98	0.85
Helicobacter pylori infection^§^	2	0.03	4	0.28	0.02	2	0.14	3	0.21	0.65

Chi-square test

^†^t-test

^§^Fisher exact test; SD = standard deviation; NTD = New Taiwan Dollars; 1 USD = 30 NTD

The incidence density rate of biliary tract disease was 13.9-fold higher in the LD cohort than in the non-LD cohort (10.2 vs. 0.71 per 1,000 person-years), with an adjusted HR of 14.2 (95% CI = 7.73–26.1) (Table 2). In the multivariable model, the risk for biliary tract disease was 1.96-fold increased for men compared with women (95% CI = 1.10–3.50) and higher for patients with rheumatism (excluding the back) (adjusted HR = 3.63, 95% CI = 1.07–12.3). In study subjects aged ≤ 35 years, patients with LD had a 15.2-fold increased risk of biliary tract disease compared with patients without LD (adjusted HR = 15.2, 95% CI = 6.91–33.3); in patients aged > 35 years, the adjusted HR of biliary tract disease was 14.0 (95% CI = 5.34–36.8) for the LD patients compared with the non-LD subjects (Table 3). In the sex-specific analysis, the incidences in the LD cohort were higher than in the non-LD one, and the risks of biliary tract disease were higher in men than in women (adjusted HR = 28.6, 95% CI = 11.9–68.4 for men; adjusted HR = 5.14, 95% CI = 1.98–13.4 for women). The monthly income analysis revealed that patients with LD, compared with patients without, exhibited a higher risk in the monthly income category of ≥NT$22,800 (adjusted HR = 23.2, 95% CI = 6.54–82.6). Overall, stratified by comorbidity, the relative risk of biliary tract disease was higher in the LD cohort than in the non-LD cohort for both patients with or without comorbidity. The incidence density rates were significantly higher in the first 3 years, 13.5 per 1,000 person-years in the biliary tract disease with the LD cohort. The highest adjusted HR of biliary tract disease for LD patients compared with the non-LD cohort was 22.4 (95% CI = 10.8–46.1) for patients with follow-up of ≤3 years.

**Table 2:** Incidence and hazard ratios for biliary tract disease and biliary tract disease–associated risk factors in the age and sex matched cohorts.

Variable	Event	PY	IR	IRR(95% CI)	Adjusted HR (95% CI)
**Donors**					
None	14	19717	0.71	1(Reference)	1(Reference)
All	42	4136	10.2	13.9(7.62, 25.5)***	14.2(7.73, 26.1)***
**Age, year**					
≤35	36	15452	2.33	1(Reference)	1(Reference)
>35	20	8401	2.38	1.04(0.60, 1.80)	1.22(0.64, 2.00)
**Sex**					
Female	17	10831	1.57	1(Reference)	1(Reference)
Male	39	13021	3.00	1.90(1.07, 3.36)*	1.96(1.10, 3.50)*
**Monthly income(NT$)**					
<15,000	18	7603	2.37	1.16(0.62, 2.16)	1.19(0.63, 2.22)
15,000–22,799	22	11052	1.99	1(Reference)	1(Reference)
≥22,800	16	5198	3.08	1.60(0.84, 3.05)	1.59(0.83, 3.05)
**Comorbidity**					
**Arthropathies and related disorders**					
No	56	23631	2.37	1(Reference)	1(Reference)
Yes	0	222	0.00	-	-
**Dorsopathies**					
No	56	23642	2.37	1(Reference)	1(Reference)
Yes	0	211	0.00	-	-
**Rheumatism, excluding the back**					
No	53	23649	2.24	1(Reference)	1(Reference)
Yes	3	204	14.7	6.40(2.00, 20.5)**	3.63(1.07, 12.3)*
**Osteopathies, chondropathies, and acquired musculoskeletal deformities**					
No	56	23617	2.37	1(Reference)	1(Reference)
Yes	0	236	0.00	-	-
**Ischemia heart disease**					
No	55	23739	2.32	1(Reference)	1(Reference)
Yes	1	113	8.82	3.87(0.54, 27.9)	1.48(0.18, 12.1)
**Diabetes mellitus**					
No	55	23661	2.32	1(Reference)	1(Reference)
Yes	1	192	5.20	2.38(0.33, 17.2)	2.01(0.26, 15.8)
**Helicobacter pylori infection**					
No	56	23845	2.35	1(Reference)	1(Reference)
Yes	0	8	0.00	-	-

Incidence of diagnosis in follow-up period in liver donors and non-donors

IR = incidence rate, IRR = incidence rate ratio, PY = per 1,000 person-years

HR = hazard ratio by multiple analysis including age; sex; monthly income arthropathies and related disorders; dorsopathies; rheumatism (excluding the back); osteopathies, chondropathies, and acquired musculoskeletal deformities; ischemic heart disease; diabetes mellitus; and helicobacter pylori infection

*p < 0.05

**p < 0.01

***p < 0.001

**Table 3:** Incidence of biliary tract disease by age, sex, income, and follow-up time and measured hazards ratios for donors compared with non-donors in the age and sex matched cohorts.

	Non-donors			Donors			Donors to Non-donors	Adjusted HR (95% CI)
Variables	Event	PY	IR	Event	PY	IR	IRR (95% CI)	
**Age, year**								
≤35	8	12566	0.64	28	2886	9.70	15.1(6.90.33.2)***	15.2(6.91, 33.3)***
>35	6	7150	0.84	14	1250	11.2	12.5(4.80, 32.6)***	14.0(5.34, 36.8)***
**Sex**								
Female	8	8832	0.91	9	1999	4.50	4.95(1.91, 12.8)**	5.14(1.98, 13.4)***
Male	6	10885	0.55	33	2137	15.4	26.5(11.1, 63.3)***	28.6(11.9, 68.4)***
**Monthly income(NTD$)**								
<15,000	6	6367	0.94	12	1236	9.70	10.1(3.78, 26.9)***	10.7(3.99, 28.7)***
15,000–22,799	5	9015	0.55	17	2036	8.35	15.0(5.54, 40.7)***	15.7(5.77, 42.6)***
≥22,800	3	4334	0.69	13	863	15.1	19.6(5.59, 68.9)***	23.2(6.54, 82.6)***
**Comorbidity**^**‡**^								
No	13	19011	0.68	38	3857	9.85	14.2(7.55, 26.6)***	14.6(7.70, 27.2)***
Yes	1	706	1.42	4	279	14.3	8.76(0.98, 78.4)	17.8(1.62, 195.1)*
**Follow-up time**								
≤3	9	14201	0.63	41	3038	13.5	20.9(10.1, 42.9)***	22.4(10.8, 46.1)***
>3	5	5516	0.91	1	1098	0.91	1.05(0.12, 8.97)	1.08(0.12, 9.53)

Incidence of diagnosis in follow-up period in liver donors and non-donors

IR = incidence rate, IRR = incidence rate ratio, PY = per 1,000 person-years

HR = hazard ratio by multiple analysis including age; sex; monthly income; arthropathies and related disorders; dorsopathies; rheumatism (excluding the back); osteopathies, chondropathies, and acquired musculoskeletal deformities; ischemic heart disease; diabetes mellitus; and helicobacter pylori infection.

Comorbidity^‡^: Patients with any one of the following comorbidities: arthropathies and related disorders; dorsopathies; rheumatism (excluding the back); osteopathies, chondropathies, and acquired musculoskeletal deformities; ischemic heart disease; diabetes mellitus; and helicobacter pylori infection, were classified as the comorbidity group.

*p < 0.05

**p < 0.01

***p < 0.001

Compared with the non-LD cohort, patients with LD were associated with a significantly higher risk of developing biliary tract diseases (adjusted HR = 49.7, 95% CI = 15.0–164.7), and patients with LD were associated with a significantly higher risk of acquiring diagnosis of cholelithiasis (adjusted HR = 5.54, 95% CI = 2.46–12.5) (Table 4).

**Table 4:** Incidence of cholelithiasis and other diseases of the biliary tract and measured hazards ratios for donors compared with non-donors in the age and sex matched cohorts.

	Non-donors			Donors			Donors to Non-donors	Adjusted HR (95% CI)
Variables	Event	PY	IR	Event	PY	IR	IRR (95% CI)	
Cholelithiasis	11	19717	0.56	13	4136	3.14	5.57(2.49, 12.4)***	5.54(2.46, 12.5)***
Other disease of biliary tract	3	19716	0.15	29	4136	7.01	44.5(13.5, 146.0)***	49.7(15.0, 164.7)***

Incidence of diagnosis in follow-up period in liver donors and non-donors

IR = incidence rate, IRR = incidence rate ratio, PY = per 1,000 person-years

HR = hazard ratio by multiple analysis including age; sex; monthly income; arthropathies and related disorders; dorsopathies; rheumatism (excluding the back); osteopathies, chondropathies, and acquired musculoskeletal deformities; ischemic heart disease; diabetes mellitus; and helicobacter pylori infection

***p < 0.001

For balancing this confounding factors, we used propensity-score matching reduce the different between two cohorts and compare the biliary tract disease, cholelithiasis, and other disease of biliary tract risk associations, which generated findings similar to those of age and sex matched cohort (Table 5).

**Table 5:** Overall biliary tract disease incidence (per 1000 person-years) and estimated HRs in donors compared with non-donors using a time-dependent regression model after propensity-score matching.

	Propensity Score Matched	
	Donors	
Variables	No(N = 1434)	Yes(N = 1434)
Biliary tract disease		
Person-years	4919	4110
Event, n	3	42
IR	0.61	10.2
IRR (95% CI)	1(Reference)	16.1(5.00, 52.0)***
Adjusted HR (95% CI)	1(Reference)	18.8(5.67, 62.2)***
Cholelithiasis		
Person-years	4919	4110
Event, n	2	13
IR	0.41	3.16
IRR (95% CI)	1(Reference)	7.50(1.69, 33.2)**
Adjusted HR (95% CI)	1(Reference)	7.50(1.69, 33.3)**
Other disease of biliary tract		
Person-years	4919	4110
Event, n	1	29
IR	0.20	7.06
IRR (95% CI)	1(Reference)	33.4(4.55, 245.1)***
Adjusted HR (95% CI)	1(Reference)	40.7(5.37, 308.0)***

Incidence of diagnosis in follow-up period in liver donors and non-donors

IR = incidence rate, IRR = incidence rate ratio, PY = per 1,000 person-years

HR = hazard ratio by multiple analysis including age; sex; monthly income; arthropathies and related disorders; dorsopathies; rheumatism (excluding the back); osteopathies, chondropathies, and acquired musculoskeletal deformities; ischemic heart disease; diabetes mellitus; and helicobacter pylori infection

**p < 0.01

***p < 0.001

## Discussion

Lei et al reported that biliary complications were the most common complications, with an incidence of 9% [11]. Ghobrial et al reviewed 405 donors, finding that 9% of them had biliary leaks [9]. Broelsch et al showed that 14.6% of living liver donors experienced biliary leak or stricture in Europe [15]. Hashikura et al reported that biliary complications occurred in 3% of living liver donors [17]. Trottet et al also found that the values of alkaline phosphatase have a slower return to baseline among living liver donors [16]. The aforementioned results indicate the immediate complications post liver donation. Following on from their results, our study demonstrated that living liver donors have a higher risk of biliary tract disease in long-term follow-up. Our data clearly show that the living liver donors had higher risks of biliary tract disease compared with age-and sex-matched controls. Thus, careful evaluation and awareness during the donation process are necessary to reduce the incidence of biliary complications among donors to zero.

Liver transplant has been reported to impair the motility of the sphincter of oddi as well as cholecystokinin response in recipients [21,22]. Rerknimitr et al used endoscopy to evaluate liver recipients; 24.5% of the patients in their study experienced biliary complications, and 8 patients had dilated recipient and donor ducts [21]. However, the motility and physiological function of the biliary tract in liver donors had never been investigated. We argue that liver resection also damages the microstructure of the biliary tract and its response to cholecystokinin in donors, thus impairing the motility of the biliary tract and increasing the risk of biliary tract disease following liver donation.

Biliary tract disease is generally more common in female donors due to pregnancy, estrogen, and contraceptives use [23,24]. However, notably, our study revealed that male donors had higher risks of biliary tract disease than did female donors. A possible explanation is that microanatomy changes following liver resection outweighed the risk factors of pregnancy, estrogen, and contraceptives use. Another possible explanation is that women suitable for liver donation had fewer of these conventional risk factors than did other women.

This study had several limitations. First, information regarding the levels of HbA1C, glucose, bilirubin, alkaline phosphatase, γ-GT, lipoprotein, and triglyceride was not available in the NHIRD. Second, no data regarding personal dietary preferences, smoking, alcohol consumption, daily activity, and body mass index were contained in the NHIRD. Third, the surgical procedure, surgical findings, and imaging study of the biliary tract are not detailed in this database. There is no available information on the type of hepatectomy the donors had undergone and if there is a different risk according to the type of resection. Fourth, it is unclear the incidence of cholelithiasis in the LD population since in most cases a cholecystectomy should have been performed. Although we considered rheumatism and dorsopathies as risk factors of biliary tract disease, several risk factors such as opiates use, multiple transfusions, chronic infections, major trauma, and total parenteral nutrition were not considered in this study. Comparison between groups may be problematic since there are major differences between the groups in regards to biliary tract disease. Fifth, we did not subclassify biliary tract disease in this study, making it difficult to investigate further the association between liver donation and biliary tract disease. Finally, we had no information about whether donors have a cholecystectomy at the time of liver donation. Therefore, the increasing incidences of cholelithiasis in LD group would be incidental findings. A code of cholelithiasis was added based on histopathology there were gall stones.

In conclusion, our study revealed that liver donation is associated with an increased risk of biliary tract disease, especially in male donors. Clinicians should be aware of these findings during imaging follow-up with living liver donors.

## Supporting information

S1 Checklist The RECORD statement–checklist of items, extended from the STROBE statement, that should be reported in observational studies using routinely collected health data. (DOCX)

## Abbreviations

LDLT: living-donor liver transplantation
NHIRD: National Health Insurance Research Database
ICD-9-CM: International Classification of Diseases, Ninth Revision, Clinical Modification
SD: standard deviation
IRR: incidence rate ratio
CI: confidence interval
HR: hazard ratio
